# Building a cancer risk and survival prediction model based on social determinants of health combined with machine learning: A NHANES 1999 to 2018 retrospective cohort study

**DOI:** 10.1097/MD.0000000000041370

**Published:** 2025-02-07

**Authors:** Shiqi Zhang, Jianan Jin, Qi Zheng, Zhenyu Wang

**Affiliations:** a Graduate School, Zhejiang Chinese Medical University, Hangzhou, Zhejiang, PR China; b State Key Laboratory of Infectious Disease Diagnosis and Treatment, The First Affiliated Hospital of Zhejiang University School of Medicine, National Clinical Research Center for Infectious Diseases, National Medical Center for Infectious Diseases, Collaborative Innovation Center for Infectious Disease Diagnosis and Treatment, Hangzhou, Zhejiang, PR China; c School of Medicine, Shaoxing University, Shaoxing, Zhejiang, PR China.

**Keywords:** cancer risk, cancer survival, machine learning, social determinants of health

## Abstract

The occurrence and progression of cancer is a significant focus of research worldwide, often accompanied by a prolonged disease course. Concurrently, researchers have identified that social determinants of health (SDOH) (employment status, family income and poverty ratio, food security, education level, access to healthcare services, health insurance, housing conditions, and marital status) are associated with the progression of many chronic diseases. However, there is a paucity of research examining the influence of SDOH on cancer incidence risk and the survival of cancer survivors. The aim of this study was to utilize SDOH as a primary predictive factor, integrated with machine learning models, to forecast both cancer risk and prognostic survival. This research is grounded in the SDOH data derived from the National Health and Nutrition Examination Survey dataset spanning 1999 to 2018. It employs methodologies including adaptive boosting, gradient boosting machine (GradientBoosting), random forest (RF), extreme gradient boosting, light gradient boosting machine, support vector machine, and logistic regression to develop models for predicting cancer risk and prognostic survival. The hyperparameters of these models—specifically, the number of estimators (100–200), maximum tree depth (10), learning rate (0.01–0.2), and regularization parameters—were optimized through grid search and cross-validation, followed by performance evaluation. Shapley Additive exPlanations plots were generated to visualize the influence of each feature. RF was the best model for predicting cancer risk (area under the curve: 0.92, accuracy: 0.84). Age, non-Hispanic White, sex, and housing status were the 4 most important characteristics of the RF model. Age, gender, employment status, and household income/poverty ratio were the 4 most important features in the gradient boosting machine model. The predictive models developed in this study exhibited strong performance in estimating cancer incidence risk and survival time, identifying several factors that significantly influence both cancer incidence risk and survival, thereby providing new evidence for cancer management. Despite the promising findings, this study acknowledges certain limitations, including the omission of risk factors in the cancer survivor survival model and potential biases inherent in the National Health and Nutrition Examination Survey dataset. Future research is warranted to further validate the model using external datasets.

## 1. Introduction

Cancer is a chronic health condition that poses increasingly severe public health and social challenges in many countries and regions. Statistics indicate that 20% of the global population is currently or will eventually be affected by cancer,^[1]^ with a notable decline in the age of onset.^[2–4]^ Approximately 1 in 9 men and 1 in 12 women ultimately die from cancer. On the other hand, patients with cancer often undergo a prolonged, multistage development process in which factors such as economic status and access to healthcare services are closely related to the course of the disease.^[5,6]^ Social determinants of health (SDOH) refer to the sum of various social, economic, and environmental conditions that impact individual and population health outcomes.^[7]^ In 2020, a report by the World Health Organization outlined 7 aspects of SDOH: economic stability (such as employment and income), educational opportunities, housing conditions, food security, healthcare access, social support (including social inclusion and nondiscrimination, as well as, neighborhood relationships), and community environment. Current research reports indicate that populations facing adverse SDOH, such as low education and income levels, exhibit higher rates of cardiovascular disease and diabetes.^[8,9]^ In addition, other studies have highlighted the significant correlation between healthy SDOH and the quality of life for patients with chronic diseases such as chronic kidney disease and diabetes.^[10]^

Current studies on the relationship between cancer and SDOH reveal a connection between SDOH and cancer screening as well as survival rates after diagnosis. A systematic review of breast cancer, cervical cancer, colorectal cancer, and lung cancer found that interventions targeting SDOH increased overall cancer screening rates by 8.4%.^[11]^ In addition, researchers have identified a correlation between residential instability and increased cervical cancer screening.^[12]^ Furthermore, an analysis by Singal et al of 32,239 patients with hepatocellular carcinoma revealed that refusal to undergo curative treatment was associated with being Black and having a lower educational level. Conversely, patients with melanoma facing adverse SDOH factors such as being unmarried, lacking insurance or private insurance, having low educational attainment, and having poor economic stability had shorter survival times.^[13,14]^ Reports on patients with breast cancer and colorectal cancer indicate that accumulating negative SDOH factors are key determinants of reduced survival times and adverse events.^[15–17]^ Despite existing studies elucidating the relationship between cancer and SDOH, it is important to note that current research primarily focuses on screening or survivorship of single cancer types, lacking a comprehensive perspective on cancer. Moreover, most studies have only explored the impact of a subset of SDOH factors on their respective cohorts. In statistical terms, traditional models often rely on linear assumptions and may not fully capture the relationship between variables. There remains a limited overall quantity of research regarding the comprehensive analysis of how all SDOH factors interact and their collective impact on overall cancer screening and survival rates of patients with cancer. In response to the current state of research, we utilize data from the National Health and Nutrition Examination Survey (NHANES) and apply machine learning techniques to investigate the relationship between SDOH and both cancer risk and survival. Machine learning, an emerging technology in the field of artificial intelligence, offers distinct advantages in analyzing complex relationships within SDOH data. It not only captures high-dimensional and nonlinear interactions among SDOH factors but also enables the examination of multiple interacting SDOH variables. At the same time, machine learning methods can handle data challenges such as missing values and class imbalances, enhancing the robustness and reliability of analyses. Furthermore, our study will adopt the social–ecological model as a theoretical framework.^[18]^ The social–ecological model emphasizes the interrelationship between individual health and environmental factors, positing that individual health is influenced by multiple SDOHs. Our study, based on this framework, highlights the impact of various SDOH factors on cancer incidence as an outcome and explores the SDOH factors that affect the survival of cancer survivors. Accordingly, we propose the following specific research questions and hypotheses: adverse SDOH factors will increase cancer incidence and mortality. Based on these research questions and hypotheses, the aim of this study was to quantify the specific effects of SDOH on cancer incidence risk and the survival rates of cancer survivors, providing decision support for effective public health interventions and a deeper understanding of cancer prevention and control efforts.

## 2. Methods

### 2.1. Data source and study population

The data for this study are sourced from the NHANES, an epidemiological survey project led by the National Center for Health Statistics of the US Department of Health and Human Services since 1998. The NHANES database includes a comprehensive collection of datasets encompassing demographic information, examination data, laboratory data, and questionnaire responses. Based on these datasets, this study collects information regarding SDOH, including employment status, family income and poverty ratio, food security, education level, access to healthcare services, health insurance, housing conditions, and marital status, along with covariate data such as age, gender, race, and body mass index (BMI). The mortality data for patients with cancer were obtained from the National Death Index match. Participants with missing data were excluded from our study. The specific inclusion and exclusion criteria are as follows: participants aged over 18 years from 1998 to 2018; participants with complete SDOH information (employment status, family income and poverty ratio, food security, education level, access to healthcare services, health insurance, housing conditions, and marital status) and covariate information (age, gender, race, and BMI); and participants with clear information regarding cancer diagnosis and cancer survivors with definitive follow-up time and survival outcomes. Exclusion criteria included missing data for any of the aforementioned variables and participants residing outside of the United States. Research involving human subjects was approved by the National Center for Health Statistics Research Ethics Review Board. These studies were conducted in accordance with local legislation and institutional requirements.

### 2.2. Assessment of SDOH and covariates SDOH

The recorded SDOH factors were assigned values of 0 for favorable SDOH and 1 for unfavorable SDOH, following the methodology of Bundy et al^[19]^ Employment status was categorized as favorable for employed individuals, students, and retirees, whereas unfavorable was assigned to unemployed individuals. Family income and poverty ratio were considered favorable if they were ≥ 300% and unfavorable if < 300%. Food security was classified as favorable if fully secure and unfavorable if marginal, low, or very low security. Educational attainment was deemed favorable for individuals with at least a high school diploma and unfavorable for those with less than a high school education. Access to healthcare services was rated favorable for individuals regularly receiving care from at least 1 formal healthcare institution, while those with no access or relying solely on emergency rooms were rated unfavorable. Health insurance type was classified as favorable if private and unfavorable if government-funded or uninsured. Housing status was categorized as favorable for homeowners and unfavorable for renters or those in other arrangements. Marital status was considered favorable for married individuals or those living with a partner, while unmarried individuals or those living with a partner were rated unfavorable. The cumulative SDOH variable was divided into 6 categories (0, 1, 2, 3, 4, and 5 or more) as shown in Tables 1 and 2. Ethnic groups were categorized based on questionnaire responses into 5 groups: Mexican, Other Hispanic, Non-Hispanic White, Non-Hispanic Black, and Other Races, with one-hot encoding applied for analysis. BMI was classified as outside the normal range (≥24) or within the normal range (<24). For handling missing data, we employed a complete-case analysis, retaining only samples with nonmissing values across all variables. Specifically, any sample with missing data in any variable was excluded entirely. This approach ensured consistency and integrity in our sample analysis, minimizing biases that could arise from missing data.

**Table 1:** Investigator characteristics.

n = 42,003	Self-reported race and ethnicity						p
	Mexican Americans (n = 6852)	Other Hispanics (n = 3341)	Non-Hispanic Whites (n = 19,143)		Non-Hispanic Blacks (n = 8837)	Other races including multiracial (n = 3830)	*P*
Age (yr)							<.001
48 (34, 63)	44 (31.0, 60.0)	47 (33.0, 61.0)	51 (35.0, 68.0)	47 (33.0, 62.0)		44 (32.0, 58.0)	
Sex							.001
Woman: 21,721 (51.71%)	3528 (51.49%)	1831 (54.80%)	9748 (50.95%)	4616 (52.23%)		1980 (51.70%)	
Man: 20,282 (48.29%)	3324 (48.51%)	1510 (45.20%)	9395 (49.10%)	4203 (47.56%)		1850 (48.30%)	
BMI							<.001
<24: 32,222 (76.71%)	<24: 5787 (84.46%)	<24: 2701 (80.84%)	<24: 14,334 (74.88%)	<24: 7112 (80.48%)		<24: 2288 (59.74%)	
≥24: 9781 (23.29%)	≥24: 1065 (15.54%)	≥24: 640 (19.16%)	≥24: 4809 (25.12%)	≥24: 1725 (19.52%)		≥24: 1542 (40.27%)	
Cancer							<.001
Yes: 3879 (9.23%)	Yes: 251 (3.67%)	Yes: 198 (5.92%)	Yes: 2727 (14.24%)	Yes: 549 (6.21%)		Yes: (4.10%)	
No: 38,125 (90.77%)	No: 6601 (96.34%)	No: 3143 (94.10%)	No: 16,420 (85.78%)	No: 8828 (99.90%)		No: (95.90%)	
Employment situation							<.001
Employed, student, or retired: 24,265 (57.77%)	Employed, student, or retired: 4250 (62.03%)	Employed, student, or retired: 2000 (59.86%)	Employed, student, or retired: 10,546 (55.12%)	Employed, student, or retired: 5061 (57.28%)		Employed, student, or retired: 2418 (63.13%)	
Out of work: 17,738 (42.23%)	Out of work: 2602 (37.97%)	Out of work: 1341 (40.13%)	Out of work: 8597 (44.93%)	Out of work: 3786 (42.84%)		Out of work: 1412 (36.87%)	
Household income and poverty rate							<.001
≥300%: 15,790 (37.59%)	≥300%: 1417 (20.68%)	≥300%: 888 (26.58%)	≥300%: 8912 (46.58%)	≥300%: 2830 (32.02%)		≥300%: 1743 (45.51%)	
<300%: 26,213 (62.41%)	<300%: 5435 (79.32%)	<300%: 2453 (73.42%)	<300%: 10,231 (53.47%)	<300%: 6007 (67.98%)		<300%: 2087 (54.49%)	
Food security							<.001
Completely safe: 30,023 (71.48%)	Completely safe: 4065 (59.33%)	Completely safe: 2011 (60.19%)	Completely safe: 15,241 (79.65%)	Completely safe: 5799 (65.62%)		Completely safe: 2907 (75.90%)	
Edge, low, or very low security: 11,980 (28.52%)	Edge, low, or very low security: 11,980 (28.52%)	Edge, low, or very low security: 1330 (39.81%)	Edge, low or, very low security: 3902 (20.39%)	Edge, low, or very low security: 3038 (34.38%)		Edge, low, or very low security: 923 (24.10%)	
Level of education							<.001
High school, graduated, or above: 35,936 (85.56%)	High school, graduated, or above: 4178 (60.97%)	High school, graduated, or above: 2633 (78.81%)	High school, graduated, or above: 17,771 (92.87%)	High school, graduated, or above: 7873 (89.09%)		High school, graduated, or above: 3481 (90.88%)	
Below high school: 6067 (14.44%)	Below high school: 2674 (39.02%)	Below high school: 708 (21.19%)	Below high school: 1372 (7.17%)	Below high school: 964 (10.91%)		Below high school: 349 (9.11%)	
Access to regular health care services							<.001
At least 1 accredited medical institution: 37,043 (88.19%)	At least 1 accredited medical institution: 5734 (83.69%)	At least 1 accredited medical institution: 2609 (78.09%)	At least 1 accredited medical institution: 17,499 (91.46%)	At least 1 accredited medical institution: 7738 (87.56%)		At least 1 accredited medical institution: 3263 (85.20%)	
No or emergency room: 4960 (11.81%)	No or emergency room: 1118 (16.31%)	No or emergency room: 532 (15.92%)	No or emergency room: 1644 (8.59%)	No or emergency room: 1099 (12.44%)		No or emergency room: 567 (14.80%)	
Health insurance type							<.001
Private: 27,251 (64.88%)	Private: 3143 (45.87%)	Private: 1971 (58.99%)	Private: 13,722 (71.71%)	Private: 5614 (63.53%)		Private: 2801 (73.13%)	
The government or none: 14,752 (35.12%)	The government or the government: 3709 (54.13%)	The government or the government: 1370 (41.00%)	The government or the government: 5424 (28.34%)	The government or the government: 3223 (36.47%)		The government or the government: 1029 (26.87%)	
Residence							<0.001
Home of one’s own: 26,242 (62.48%)	Home of one’s own: 3980 (58.09%)	Home of one’s own: 3980 (58.09%)	Home of one’s own: 13,950 (72.91%)	Home of one’s own: 4484 (50.74%)		Home of one’s own: 2207 (57.62%)	
Rent a house or other arrangement: 15,761 (37.52%)	Rent a house or other arrangement: 2872 (41.91%)	Rent a house or other arrangement: 2872 (41.91%)	Rent a house or other arrangement: 5193 (27.14%)	Rent a house or other arrangement: 4353 (49.26%)		Rent a house or other arrangement: 1623 (42.38%)	
Marital status							<.001
Married or living together with your partner: 26,384 (62.81%)	Married or living together with your partner: 4832 (70.52%)	Married or living together with your partner: 2092 (62.62%)	Married or living together with your partner: 12,579 (65.74%)	Married or living together with your partner: 4243 (48.01%)		Married or living together with your partner: 2638 (68.88%)	
Unmarried or live together with your partner: 15,619 (37.18%)	Unmarried or live together with your partner: 2020 (29.48%)	Unmarried or live together with your partner: 1249 (37.38%)	Unmarried or live together with your partner: 6564 (34.31%)	Unmarried or live together with your partner: 4594 (51.99%)		Unmarried or live together with your partner: 1192 (31.12%)	
SDOH variables were accumulated							<.001
0: 5263 (12.53%)	0: 383 (5.59%)	0: 269 (8.05%)	0: 3266 (17.07%)	0: 757 (8.57%)		0: 588 (15.35%)	
1: 7198 (17.14%)	1: 757 (11.05%)	1: 371 (11.10%)	1: 4087 (21.36%)	1: 1204 (13.62%)		1: 779 (20.34%)	
2: 7470 (17.78%)	2: 953 (13.91%)	2: 551 (16.49%)	2: 3803 (19.88%)	2: 1430 (16.18%)		2: 733 (19.14%)	
3: 7698 (18.33%)	3: 1327 (19.37%)	3: 628 (18.80%)	3: 3402 (17.78%)	3: 1692 (19.15%)		3: 649 (16.95%)	
4: 6975 (16.61%)	4: 1485 (16.61%)	4: 641 (19.18%)	4: 2552 (13.34%)	4: 1709 (19.34%)		4: 588 (15.35%)	
5: 4873 (11.60%)	5: 1194 (17.43%)	5: 562 (16.82%)	5: 1459 (7.63%)	5: 1321 (14.95%)		5: 337 (8.80%)	
More than 6 points: 2526 (6.01%)	More than 6 points: 753 (10.99%)	More than 6 points: 319 (9.55%)	More than 6 points: 574 (3.00%)	More than 6 points: 688 (7.79%)		More than 6 points: 156 (4.07%)	

The Mann–Whitney *U* analysis was used for continuous variables. Categorical variables were analyzed using the χ^2^ test. A disadvantage with a score of 1: out of work, household income, poverty rate <300%, edge, low, or very low food security, below high school, no regular medical care or only visits to the emergency room, government insurance or no insurance, rent a house or other arrangement, unmarried or live together with your partner.Favorable factors with a score of 0: employment situation, employed, student, or retired.Household income and poverty rate ≥ 300%: food security completely safe, high school, graduated, or above, at least 1 accredited medical institution, private health insurance, home of one’s own, married or living together with your partner.

BMI = body mass index, SDOH = social determinants of health.

**Table 2:** Baseline table stratified by cancer.

n = 42,003	Patients were grouped according to whether they had cancer		
	No cancer was diagnosed (n = 38,125)	A cancer diagnosis (n = 3878)	*P*
Age (yr)			<.001
48 (34, 63)	47.3 (17.4)	65.2 (14.6)	
Sex			.105
Woman: 21,721 (51.71%)	Woman: 19,667 (51.6%)	Woman: 2054 (53.0%)	
Man: 20,282: (48.29%)	Man: 18,458 (48.4%)	Man: 1824 (47.0%)	
BMI			.115
<24: 32,222 (76.71%)	<24: 29,207 (76.6%)	<24: 3015 (77.7%)	
≥24: 9781 (23.29%)	≥24: 8918 (23.4%)	≥24: 863 (22.3%)	
Race			<.001
Mexican: n = 6852	Mexican: 6601 (17.3%)	Mexican: 251 (6.47%)	
Non-Hispanic Blacks: n = 8837	Non-Hispanic Blacks: 8288 (21.7%)	Non-Hispanic Blacks: 549 (14.2%)	
Non-Hispanic Whites: n = 19,143	Non-Hispanic Whites: 16,420 (43.1%)	Non-Hispanic Whites: 2723 (70.2%)	
Other Hispanics: n = 3341	Other Hispanics: 3143 (8.24%)	Other Hispanics: 198 (5.11%)	
The other races: n = 3830	The other races: 3673 (9.63%)	The other races: 157 (4.05%)	
The employment situation			<.001
Employed, student, or retired: 24,265 (57.77%)	Employed, student, or retired: 23,054 (60.5%)	Employed, student, or retired: 1211 (31.2%)	
Out of work: 17,738 (42.23%)	Out of work: 15,071 (39.5%)	Out of work: 2667 (68.8%)	
Household income and poverty rate			<.001
≥300%: 15,790 (37.59%)	≥300%: 14,135 (37.1%)	≥300%: 1655 (42.7%)	
<300%: 26,213 (62.41%)	<300%: 23,990 (62.9%)	<300%: 2223 (57.3%)	
Food security			<.001
Completely safe: 30,023 (71.48%)	Completely safe: 26,980 (70.8%)	Completely safe: 3043 (78.5%)	
Edge, low, or very low security: 11,980 (28.52%)	Edge, low, or very low security: 11,145 (29.2%)	Edge, low, or very low security: 835 (21.5%)	
Level of education			<.001
High school, graduated, or above: 35,936 (85.56%)	High school, graduated, or above: 32,542 (85.4%)	High school, graduated, or above: 3394 (87.5%)	
Below high school: 6067 (14.44%)	Below high school: 5583 (14.6%)	Below high school: 484 (12.5%)	
Access to regular health care services			<.001
At least 1 accredited medical institution: 37,043 (88.19%)	At least 1 accredited medical institution: 33,328 (87.4%)	At least 1 accredited medical institution: 3715 (95.8%)	
No or emergency room: 4960 (11.81%)	No or emergency room: 4797 (12.6%)	No or emergency room: 163 (4.20%)	
Health insurance type			<.001
Private: 27,251 (64.88%)	Private: 24,394 (64.0%)	Private: 2857 (73.7%)	
The government or none: 14,752 (35.12%)	The government or none: 13,731 (36.0%)	The government or none: 1021 (26.3%)	
Residence			<.001
Home of one’s own: 26,242 (62.48%)	Home of one’s own: 23,247 (61.0%)	Home of one’s own: 2995 (77.2%)	
Rent a house or other arrangement: 15,761 (37.52%)	Rent a house or other arrangement: 14,878 (39.0%)	Rent a house or other arrangement: 883 (22.8%)	
Marital status			.085
Married or living together with your partner: 26,384 (62.81%)	Married or living together with your partner: 23,998 (62.9%)	Married or living together with your partner: 2386 (61.5%)	
Unmarried or live together with your partner: 15,619 (37.18%)	Unmarried or live together with your partner: 14,127 (37.1%)	Unmarried or live together with your partner: 1492 (38.5%)	
SDOH variables were accumulated			<.001
0: 5263 (12.53%)	0: 4878 (12.8%)	0: 385 (9.93%)	
1: 7198 (17.14%)	1: 6415 (16.8%)	1: 786 (20.3%)	
2: 7470 (17.78%)	2: 6595 (17.3%)	2: 880 (22.7%)	
3: 7698 (18.33%)	3: 6948 (18.2%)	3: 759 (19.6%)	
4: 6975 (16.61%)	4: 6417 (16.8%)	4: 568 (14.6%)	
5: 4873 (11.60%)	5: 4506 (11.8%)	5: 356 (9.18%)	
More than 6 points 2526 (6.01%)	More than 6 points 2366 (6.21%)	More than 6 points 144 (3.71%)	

Variables include demographic details, socioeconomic factors, and health-related metrics. Continuous variables were analyzed using the Mann–Whitney *U* test, while categorical variables were analyzed using the χ^2^ test.

BMI = body mass index, SDOH = social determinants of health.

### 2.3. Statistical analysis

For the descriptive statistics of this study (Tables 1 and 2), continuous variables that follow a normal distribution are reported as means and standard deviations, while nonnormally distributed continuous variables are presented as medians with interquartile ranges. Categorical variables are expressed as percentages. Continuous variables were analyzed using the Mann–Whitney *U* test, and categorical variables were assessed using the χ^2^ test. In this study, we conducted a power analysis to verify the adequacy of the sample size. Based on the expected effect size (Cohen *d* = 0.5), significance level (*α* = 0.05), and target power (1 − *β* = 0.80), the required sample size for each group was calculated to be 64. Our dataset contains 42,003 samples, which fully meets the statistical power requirements. All statistical analyses were performed using R software (version 4.4.1), developed by the R Foundation for Statistical Computing, Vienna, Austria, and Python (version 3.12), developed by the Python Software Foundation, Beaverton, OR.

### 2.4. Machine learning modeling

**Figure 1.**
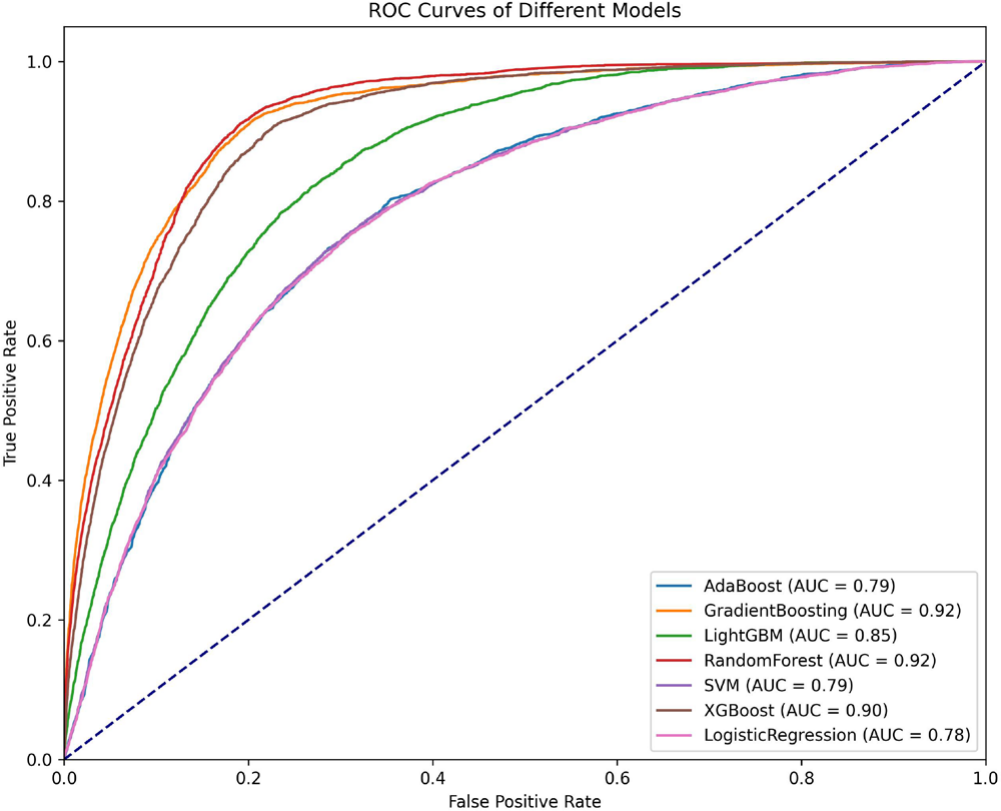
ROC curve of the cancer risk model. ROC curves of various machine learning models used to predict cancer risk. The models included are AdaBoost, gradient boosting, LightGBM, random forest, SVM, XGBoost, and logistic regression. The AUC values for each model are presented in the legend, with random forest and gradient boosting achieving the highest AUCs of 0.92, indicating superior performance in predicting cancer risk. The diagonal dashed line represents the performance of a random classifier (AUC = 0.5) for comparison. AdaBoost = adaptive boosting, AUC = area under the curve, LightGBM = light gradient boosting machine, ROC = receiver operating characteristic, SVM = support vector machine, XGBoost = extreme gradient boosting.

**Figure 2.**
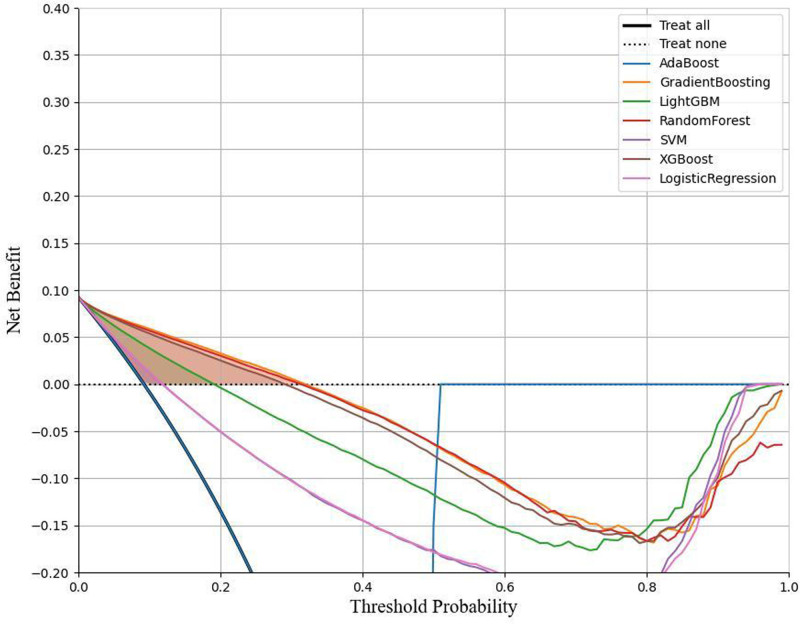
DCA curve of the cancer risk model. DCA curves for various machine learning models predicting cancer risk. The models compared include AdaBoost, gradient boosting, LightGBM, random forest, SVM, XGBoost, and logistic regression. The *y*-axis represents the net benefit, and the *x*-axis indicates the threshold probability. The “Treat all” (solid line) and “Treat none” (dotted line) strategies are included for reference, representing the net benefit of treating all or no patients, respectively, across different threshold probabilities. A model that achieves a higher net benefit across a range of threshold probabilities demonstrates better clinical usefulness. In this analysis, gradient boosting and random forest models show relatively higher net benefits at various threshold probabilities, indicating their potential utility in clinical decision-making for cancer risk assessment. AdaBoost = adaptive boosting, DCA = decision curve analysis, LightGBM = light gradient boosting machine, SVM = support vector machine, XGBoost = extreme gradient boosting.

**Figure 3.**
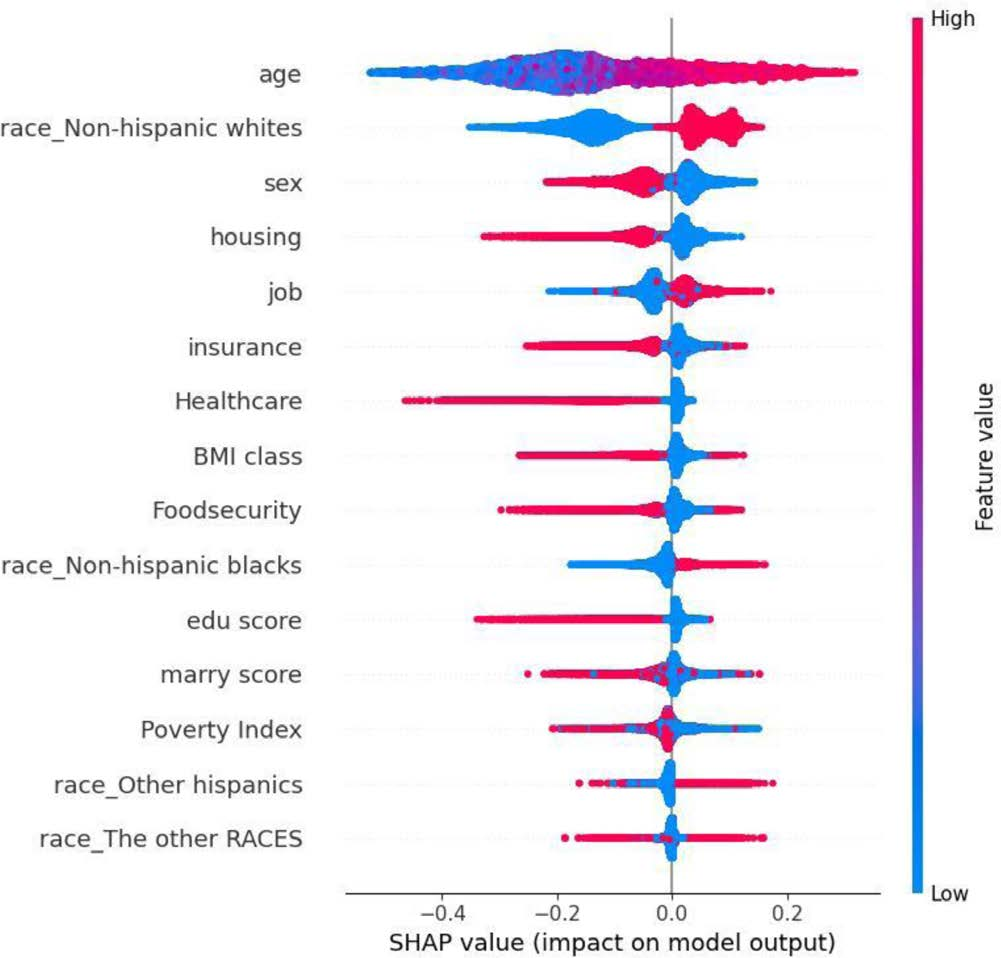
Plot of SHAP values for cancer risk. SHapley additive explanation values for the various features in the cancer risk prediction model, showing the effect of each feature on the model output. The *y*-axis lists characteristics such as age, race, sex, and socioeconomic indicators such as insurance, housing, and food security. The *x*-axis represents SHAP values, indicating the magnitude and direction of the impact of each feature on cancer risk prediction. Each point represents a separate data point and is colored to indicate the feature values (pink high and blue low). Features with higher SHAP values have a greater impact on model predictions. Among them, age seems to be a prominent factor, and a higher value of age is helpful for cancer risk prediction. This figure reveals significant effects of age, race, especially non-Hispanic Whites, and socioeconomic factors such as gender and housing situation on cancer risk. SHAP = Shapley Additive exPlanations.

The total population included in this study was randomly divided into training and validation sets in an 80:20 ratio. To predict cancer risk and mitigate the effects of data imbalance, the samples were initially subjected to resampling. Seven machine learning algorithms were employed to construct classification models: adaptive boosting (AdaBoost), gradient boosting machine (GBM), random forest (RF), extreme gradient boosting (XGBoost), light gradient boosting machine (LightGBM), support vector machine (SVM), and logistic regression. AdaBoost improves model performance by dynamically adjusting sample weights and combining weak classifiers while also indirectly reducing the risk of overfitting by increasing the weight of misclassified samples. GBM and its variant XGBoost utilize regularization techniques to effectively control model complexity and prevent overfitting. RF is renowned for its capability to optimize feature selection, significantly enhancing the model’s generalization ability. LightGBM demonstrates efficient training advantages when handling large-scale data and categorical variables due to its histogram-based algorithm. SVM minimizes the risk of overfitting by maximizing the classification margin while also exhibiting excellent resilience to background noise. Logistic regression effectively predicts the probability of event occurrence at a lower computational cost. Once the models were established, cross-validation and grid parameter tuning were employed to identify the model with optimal performance. The cross-validation method used was *k*-fold (*K* = 5), which ensures model robustness by training and validating across different folds. The grid tuning parameters were set as follows: AdaBoost (learning rate = 1.0, estimators = 100, random state = 42), GBM (learning rate = 0.2, estimators = 100, random state = 42, max depth = 10), LightGBM (learning rate = 0.1, num leaves = 31, max depth = 10, estimators = 200), RF (max depth = 20, max features = sqrt, estimators = 100, random state = 42), SVM (*C* = 1, random state = 42), XGBoost (learning rate = 0.1, max depth = 10, estimators = 100, random state = 42), K-nearest neighbors (neighbors = 10, weights = distance), and logistic regression (*C* = 1, max iter = 1000, random state = 42). To evaluate model stability and decision-making benefits, receiver operating characteristic (ROC) curves and decision curve analysis (DCA) curves were plotted (Figs. 1 and 2). Various performance metrics were calculated to validate model performance, including positive predictive value (PPV), negative predictive value (NPV), sensitivity, specificity, Matthews correlation coefficient (MCC), area under the curve (AUC), Kappa, Brier score, accuracy, and F1 score (Table 3). In addition, Shapley Additive exPlanations (SHAP) value summary plots were created to visualize the impact of the top 15 features (Fig. 3).

**Table 3:** Evaluation indicators of risk prediction models.

Method	Sensitivity	Specificity	Positive predictive value	Negative predictive value	Matthews correlation coefficient	AUC	Kappa	Brier score	F1 score	Accuracy
AdaBoost	0.67 (0.66–0.69)	0.77 (0.76–0.79)	0.22 (0.21–0.23)	0.96 (0.95–0.97)	0.28 (0.26–0.30)	0.79 (0.71–0.73)	0.22 (0.21–0.25)	0.25 (0.23–0.26)	0.33 (0.33–0.35)	0.76 (0.75–0.77)
Gradient boosting machine	0.85 (0.82–0.86)	0.84 (0.83–0.85)	0.36 (0.32–0.37)	0.98 (0.97–0.99)	0.48 (0.44–0.49)	0.92 (0.88–0.93)	0.43 (0.39–0.44)	0.10 (0.09–0.12)	0.50 (0.4–0.51)	0.84 (0.83–0.85)
RF	0.86 (0.84–0.87)	0.84 (0.83–0.85)	0.36 (0.32–0.37)	0.98 (0.97–0.99)	0.49 (0.44–0.50)	0.92 (0.90–0.95)	0.43 (0.41–0.44)	0.10 (0.09–0.17)	0.51 (0.48–0.53)	0.84 (0.83–0.85)
XGBoost	0.82 (0.80–0.83)	0.83 (0.81–0.84)	0.33 (0.32–0.34)	0.98 (0.97–0.99)	0.45 (0.41–0.46)	0.90 (0.86–0.93)	0.40 (0.36–0.42)	0.11 (0.10–0.13)	0.48 (0.44–0.52)	0.83 (0.82–0.84)
LightGBM	0.73 (0.72–0.74)	0.80 (0.79–0.81)	0.27 (0.25–0.28)	0.97 (0.96–0.98)	0.35 (0.33–0.38)	0.85 (0.80–0.89)	0.30 (0.29–0.31)	0.14 (0.12–0.16)	0.39 (0.37–0.40)	0.79 (0.78–0.80)
SVM	0.70 (0.69–0.73)	0.73 (0.70–0.74)	0.21 (0.20–0.23)	0.96 (0.95–0.97)	0.28 (0.26–0.30)	0.79 (0.77–0.81)	0.22 (0.21–0.23)	0.19 (0.17–0.21)	0.33 (0.32–0.34)	0.72 (0.71–0.74)
LogisticRegression	0.70 (0.69–0.72)	0.73 (0.72–0.74)	0.21 (0.20–0.23)	0.96 (0.95–0.97)	0.27 (0.25–0.30)	0.78 (0.77–0.80)	0.21 (0.20–0.23)	0.18 (0.16–0.20)	0.32 (0.31–0.34)	0.73 (0.72–0.74)

The evaluation metrics for various machine learning models used to predict cancer risk, including sensitivity, specificity, positive predictive value, negative predictive value, Matthews correlation coefficient, AUC, kappa, Brier score, F1 score, and accuracy. Values in parentheses represent the 95% confidence intervals.

AdaBoost = adaptive boosting, AUC = area under the curve, GradientBoosting = gradient boosting machine, LightGBM = light gradient boosting machine, LogisticRegression = logistic regression, RF = random forest, SVM = support vector machine, XGBoost = extreme gradient boosting.

**Figure 4.**
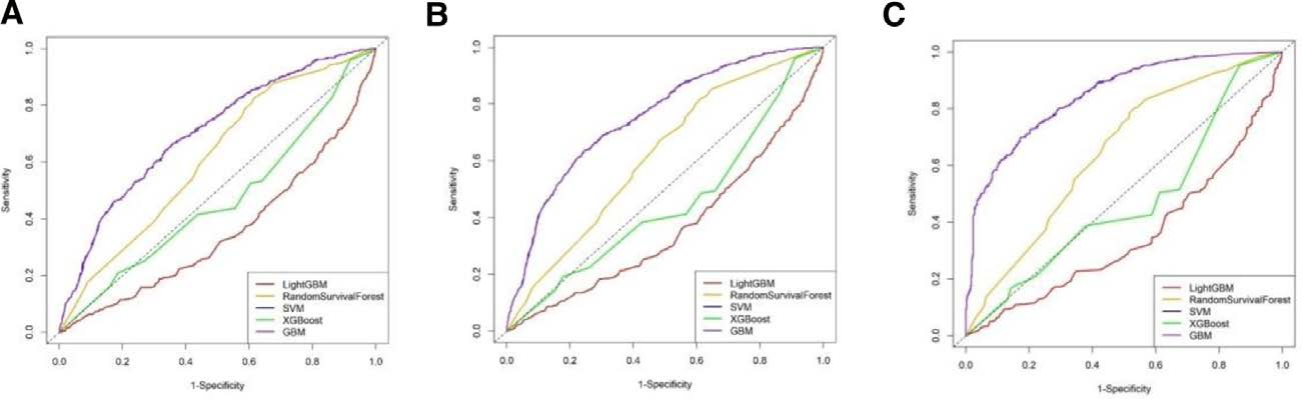
ROC curves for predictive models at different time points. ROC curves for multiple predictive models were evaluated at 3 different time points: 43, 87, and 174 mo. The curves illustrate the models’ sensitivity and specificity in predicting outcomes over time. (A) ROC curves at 43 months for the LightGBM (red), random survival forest (yellow), SVM (green), XGBoost (blue), and GBM (purple) models. The curves demonstrate the performance of each model, highlighting LightGBM’s sensitivity at lower specificity. (B) ROC curves at 87 mo, maintaining the same color coding for each model. (C) ROC curves at 174 mo, again using the same color scheme. GBM = gradient boosting machine, ROC = receiver operating characteristic, LightGBM = light gradient boosting machine, SVM = support vector machine, XGBoost = extreme gradient boosting.

**Figure 5.**
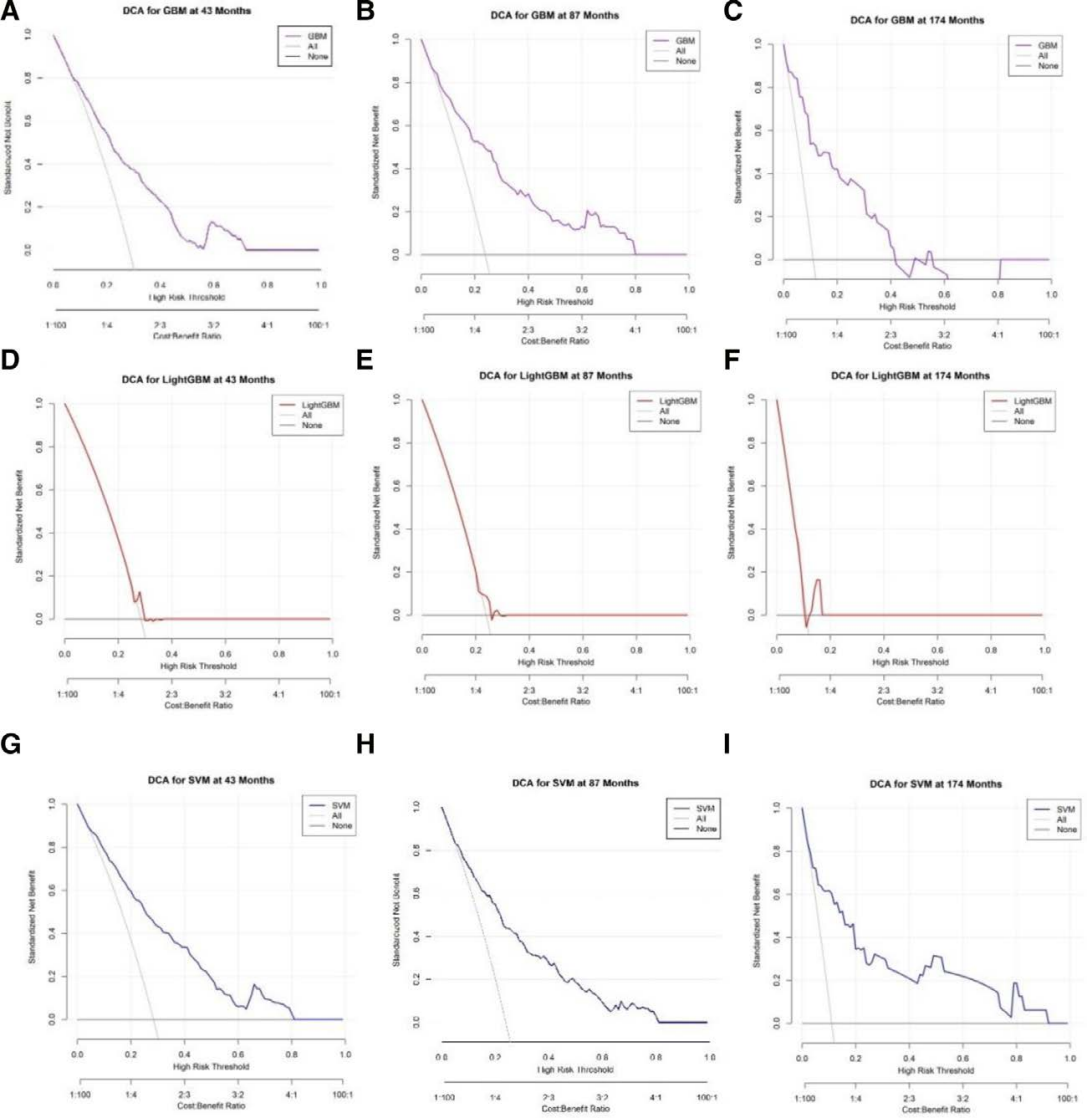
DCA for different models and time periods. DCA of various prediction models applied to survival data at different time points. Each figure illustrates the net benefit of the respective model compared to the all-or-nothing strategy over the threshold range. (A–C) DCA results for GBM at 43, 87, and 174 mo, respectively. The net benefit is represented in purple, showing the performance of the GBM model over time. (D–F) DCA results for LightGBM at 43, 87, and 174 mo, respectively. The net benefit is indicated in red, with the model’s efficacy evaluated across different risk thresholds. (G–I) DCA results for SVM at 43, 87, and 174 mo, respectively. The net benefit is depicted in blue, illustrating the model’s performance in predicting outcomes over time. DCA = decision curve analysis, GBM = gradient boosting machine, LightGBM = light gradient boosting machine, SVM = support vector machine.

**Figure 6.**
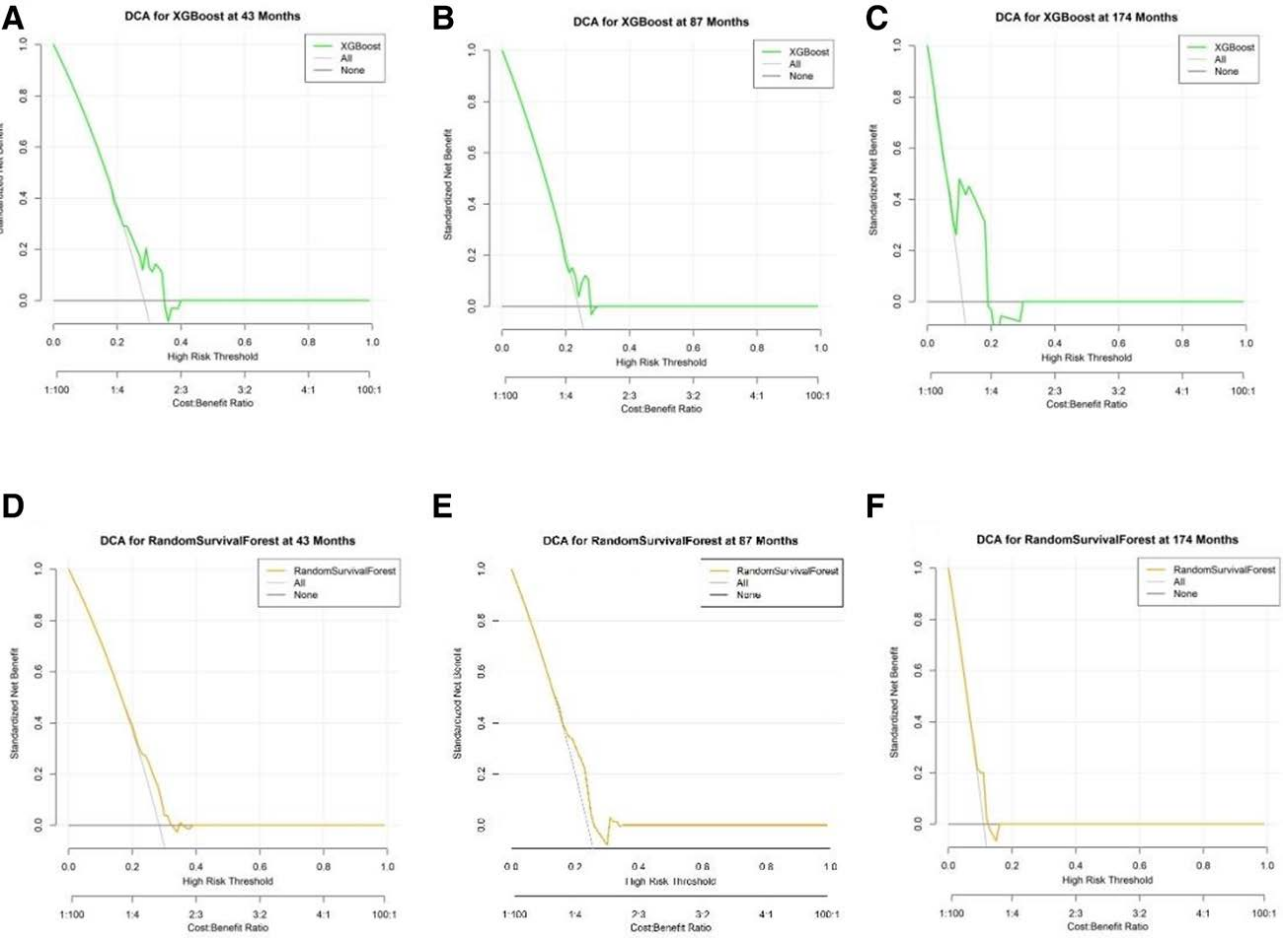
DCA for XGBoost and random survival forest models. DCA results for XGBoost and random survival forest models at various time intervals, evaluating their clinical utility in predicting survival outcomes. (A–C) DCA results for the XGBoost model at 43, 87, and 174 mo, respectively. The net benefit is shown in green, indicating the model’s performance across different high-risk thresholds. The “None” strategy is represented as a reference line for comparison. (D–F) DCA results for the random survival forest model at 43, 57, and 174 mo, respectively. The net benefit is depicted in yellow, demonstrating how the model performs over time against the “None” strategy and an “All” strategy for reference. DCA = decision curve analysis, XGBoost = extreme gradient boosting.

**Figure 7.**
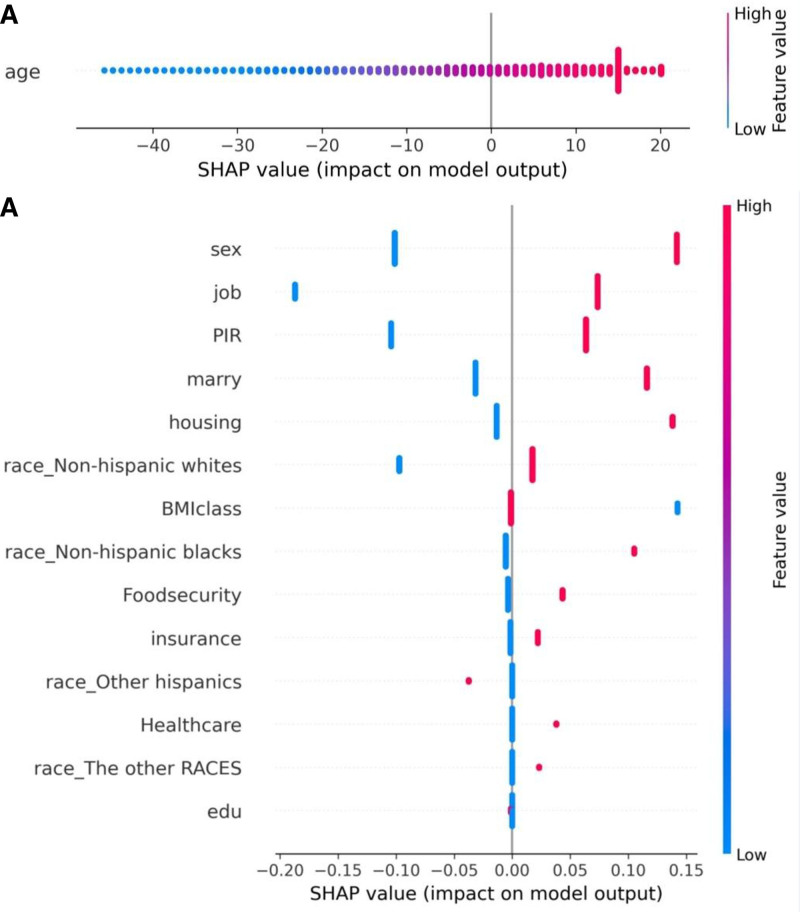
SHAP values for features impacting cancer prognosis. SHAP values, which indicate the impact of different features on the model’s output regarding cancer prognosis. (A) The distribution of SHAP values for the feature “age.” The color gradient from blue to red signifies the feature value, where blue represents lower age values and red indicates higher age values. The *x*-axis represents the SHAP values, showing the extent to which age influences the model’s predictions, with higher values correlating with a more significant positive impact on cancer prognosis. (B) SHAP values for multiple features impacting cancer prognosis. BMI = body mass index, PIR = poverty-income ratio, SHAP = Shapley Additive exPlanations.

Five machine learning algorithms were employed to construct survival models: random survival forests, support vector machine for survival analysis (SVM_Surv), gradient boosting machine for survival analysis (GBM_Surv), extreme gradient boosting for survival analysis (XGBoost_Surv), and light gradient boosting machine for survival analysis (LightGBM_Surv). These algorithms retain their original advantages while extending their application to survival analysis, ensuring good predictive performance on survival data. Cross-validation was conducted using a 5-fold approach, and grid parameter tuning was utilized to determine the model with optimal performance. The grid tuning parameters were set as follows: LightGBM_Surv (learning_rate = 0.1, max_depth = 3, num_leaves = 31), random survival forests (estimators = 200, max_features = sqrt, min_samples_leaf = 5, min_samples_split = 5), SVM_Surv (*α* = 0.01, max_iter = 1000, random_state = 42, rank_ratio = 1.0), and XGBoost_Surv (learning_rate = 0.01, estimators = 200, max_depth = 5). The median survival time was calculated, and based on this median time, multitime point ROC curves and DCA curves were plotted to evaluate model stability and decision-making benefits (Figs. 4–6). The *C*-index and mean AUC index were computed to validate model performance (Table 4), and SHAP value summary plots were created to visualize the impact of the top 15 features (Fig. 7).

**Table 4:** Evaluation indicators of survival prediction model.

Method	*C*-index	Mean AUC
RandomForest	0.75 (0.73–0.78)	0.80 (0.77–0.83)
SVM_Surv	0.75 (0.73–0.78)	0.80 (0.77–0.84)
GBM_Surv	0.75 (0.72–0.78)	0.80 (0.78–0.83)
XGBoost_Surv	0.56 (0.53–0.60)	0.49 (0.45–0.52)
LightGBM_Surv	0.43 (0.40–0.47)	0.50 (0.46–0.55)

The evaluation metrics, including mean AUC and *C*-index, for the various machine learning models used to predict cancer risk. Values in parentheses indicate 95% confidence intervals.

AUC = area under the curve, GBM_Surv = gradient boosting machine for survival analysis, LightGBM_Surv = light gradient boosting machine for survival analysis, SVM_Surv = support vector machine for survival analysis, XGBoost_Surv = extreme gradient boosting for survival analysis.

PPV and sensitivity measure the model’s ability to identify positive samples, while NPV and specificity assess the model’s ability to recognize negative samples. Higher values of these metrics indicate stronger identification capabilities. The MCC summarizes all categories in the confusion matrix and ranges from −1 to 1; a higher MCC value reflects a better predictive performance of the model. The AUC represents the area under the ROC curve, while the Kappa coefficient reports the consistency between predicted classifications and actual classifications. Larger values of the Kappa coefficient indicate more stable model performance. The Brier score is the mean of the squared differences between actual outcomes and predicted probabilities. A smaller Brier score signifies that the predicted probabilities are closer to the actual results. The F1 score is the harmonic mean of precision and recall. Accuracy represents the proportion of correct samples among the total samples. Higher values of both F1 score and accuracy indicate better model performance. The machine learning modeling and validation were conducted using Python (version 3.12).

## 3. Results

### 3.1. Population

A total of 42,003 participants were included in this study, with baseline characteristics presented in Table 1. Among them, there were 6852 (16.31%) Mexican Americans, 3341 (8.0%) Hispanic Americans, 19,143 (45.58%) non-Hispanic Whites, 8837 (21.04%) non-Hispanic Blacks, and 3830 (9.11%) individuals of other racial backgrounds. The cohort consisted of 21,721 (51.71%) females and 20,282 (48.29%) males, with a median age of 48 years (interquartile range: 34–63). Among the participants, 3879 (9.23%) reported having cancer, while 38,125 (90.77%) were cancer-free. Baseline characteristics stratified by cancer status are shown in Table 2. In this population, having a family income-to-poverty ratio of < 300% was the most commonly observed adverse SDOH factor. The most frequent cumulative SDOH score was 3. For patients with cancer, unemployment was the most frequently occurring adverse SDOH factor, with the most common cumulative SDOH score being 2. According to the baseline characteristics stratified by cancer status (Table 2), the number of patients with cancer was lower than that of patients without cancer. The average age of patients with cancer (65.2 years) was significantly higher than that of patients without cancer (47.3 years). In addition, the proportion of individuals with poor marital status among patients with cancer (38.5%) was higher than that of patients without cancer (37.1%), and the unemployment rate among patients with cancer (68.8%) was markedly greater than that of patients without cancer. Furthermore, the proportions of patients with cancer with cumulative SDOH scores of 1, 2, and 3 were also higher than those of patients without cancer.

### 3.2. Development and performance of the cancer risk model

The risk model developed in this study was evaluated through a comprehensive analysis of the ROC curve, DCA curve, and various assessment metrics indicating that the RF model exhibited the best performance (Table 3). The performance metrics for each model are as follows:

Sensitivity: RF (0.86) > GBM (0.85) > XGBoost (0.82) > LightGBM (0.73) > SVM = logistic regression (0.70) > AdaBoost (0.66).

Specificity: RF = GBM (0.84) > XGBoost (0.83) > LightGBM (0.80) > AdaBoost (0.77) > SVM = logistic regression (0.73).

PPV: RF = GBM (0.36) > XGBoost (0.33) > LightGBM (0.27) > AdaBoost (0.22) > SVM = logistic regression (0.21).

NPV: RF = GBM = XGBoost (0.98) > LightGBM (0.97) > SVM = Logistic Regression = AdaBoost (0.96).

MCC: RF (0.49) > GBM (0.48) > XGBoost (0.45) > LightGBM (0.35) > AdaBoost = SVM (0.28) > logistic regression (0.27).

AUC: RF = GBM (0.92) > XGBoost (0.90) > LightGBM (0.85) > SVM = AdaBoost (0.79) > logistic regression (0.78).

Kappa statistic: Kappa: RF = GBM (0.43) > XGBoost (0.40) > LightGBM (0.30) > SVM = AdaBoost (0.22) > logistic regression (0.21).

Brier score: Brier: AdaBoost (0.25) > SVM (0.19) > logistic regression (0.18) > LightGBM (0.14) > XGBoost (0.11) > RF = GBM (0.10).

F1 score: F1: RF (0.51) > GBM (0.50) > XGBoost (0.48) > LightGBM (0.39) > SVM = AdaBoost (0.33) > logistic regression (0.32).

Accuracy: RF = GBM (0.84) > XGBoost (0.83) > AdaBoost = LightGBM (0.79) > logistic regression (0.73) > SVM (0.72).

**Figure 8.**
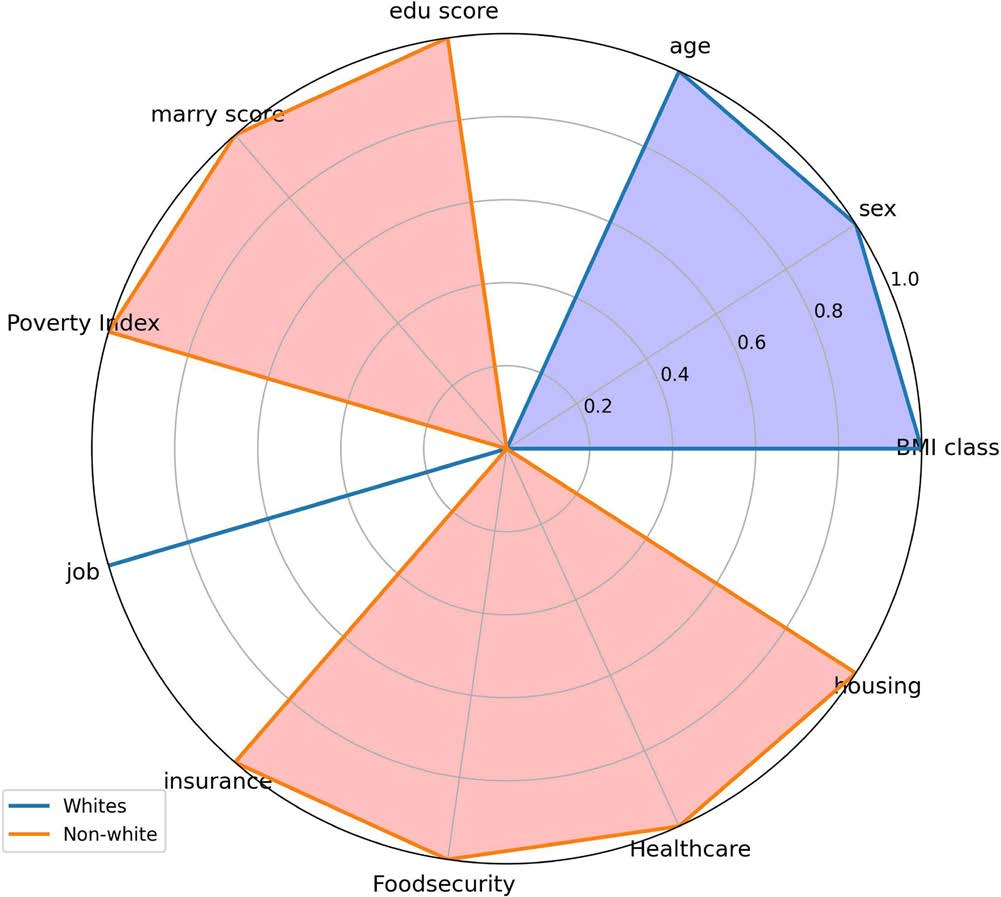
Radar chart of social determinants of health by race. The radar chart compares the social determinants of health between Whites and non-Whites across various factors. Each axis represents a specific determinant, allowing for a visual assessment of disparities between the two groups. BMI = body mass index.

**Figure 9.**
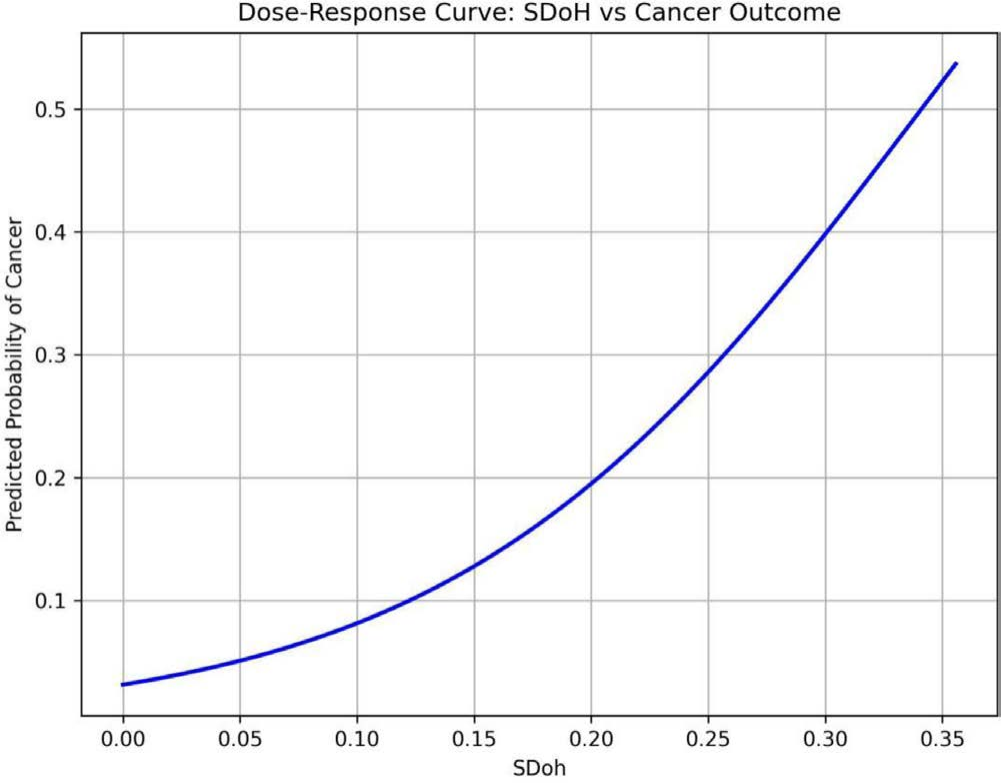
Dose–response curve. The curve reflects the cumulative effect of adverse SDOH on cancer risk. SDOH = social determinants of health.

The summary SHAP value plot based on the RF model (Fig. 3) reveals that age (mean: 48.9682, confidence interval [CI]: 48.7954–49.1385), non-Hispanic White ethnicity (mean: 0.4558, CI: 0.4508–0.4605), gender (mean: 0.4829, CI: 0.4781–0.4876), and housing status (mean: 0.3752, CI: 0.3706–0.3798) are significant features influencing the RF model. In addition, the SHAP values indicate that age, employment status, non-Hispanic Black ethnicity, poverty index, other Hispanic ethnicity, and other races predominantly have negative SHAP values, suggesting that younger individuals with stable employment, non-Hispanic Black or other Hispanic ethnicity, and better economic conditions exhibit a lower risk of developing cancer. Conversely, individuals identified as non-Hispanic White, male, with unstable housing situations, unstable insurance, unstable access to healthcare, higher BMI, food insecurity, and unstable marital status primarily have positive SHAP values, indicating that these conditions are associated with a higher cancer risk. The radar plot (Fig. 8) illustrates that non-Hispanic White individuals exhibit significantly higher scores across health-social determinants, including education, marital status, employment, insurance, food security, healthcare access, and housing compared with other racial groups. In addition, the White population demonstrates a higher average age and elevated BMI. Based on the RF model, the dose–response curve describing the relationship between health social determinants and cancer outcomes shows that cancer risk increases with the accumulation of adverse SDOH (Fig. 9).

### 3.3. Development and performance of the cancer prognostic model

A comprehensive analysis of the ROC curve, DCA curve, and model evaluation metrics indicates that the GBM is the most effective model for predicting cancer survival time. The performance metrics for each model are as follows:

*C*-index: GBM_Surv (0.75) = SVM_Surv (0.75) = RF (0.75) > XGBoost_Surv (0.56) > LightGBM_Surv (0.43).

Mean AUC: GBM_Surv (0.80) = SVM_Surv (0.80) = RF (0.80) > LightGBM_Surv (0.50) > XGBoost_Surv (0.49).

The 4 most significant features in the GBM model are age (mean: 65.1597, CI: 64.7041–65.6126), gender (mean: 0.4705, CI: 0.4547–0.4865), employment status (mean: 0.6879, CI: 0.6737–0.7026), and the family income-to-poverty ratio (mean: 0.5731, CI: 0.5574–0.5883) (Fig. 7). According to the SHAP value plot, higher values for age, non-Hispanic White ethnicity, male gender, unstable housing conditions, unstable insurance, unstable access to healthcare, higher BMI, food insecurity, and unstable marital status are associated with positive SHAP values, indicating that cancer survivors with these characteristics tend to have shorter survival times. Conversely, features such as stable employment, a higher income-to-poverty ratio, other Hispanic ethnicity, other racial backgrounds, and higher educational attainment are associated with negative SHAP values, suggesting that cancer survivors with these characteristics tend to have longer survival times.

## 4. Discussion

In the realm of research on SDOH, a 2015 health survey involving 45 states in the United States demonstrated that socioeconomic factors and health behaviors are primary contributors to population health outcomes.^[20]^ As a composite of medical security and living conditions, SDOH are closely linked to the occurrence and progression of cancer. Research has indicated that integrating SDOH into screening processes can aid in the early diagnosis and treatment of patients with cancer, as well as improve health outcomes.^[21]^ This study developed and validated cancer risk and prognostic models based on machine learning that incorporate SDOH, analyzing their impact on cancer risk and survival outcomes. The results indicated that models constructed using RF and GBM_Surv algorithms demonstrated optimal performance. SHAP values helped elucidate the significant features influencing the model; factors such as older age, non-Hispanic White ethnicity, male gender, and unstable housing conditions were associated with a higher risk of cancer. Conversely, older age, male gender, unstable employment, and a lower income-to-poverty ratio were identified as important determinants of poor survival prognosis for cancer survivors. The hypothesis of this study posited that SDOH would significantly impact cancer risk and survival rates. The findings confirm that adverse social determinants of health are positively correlated with cancer incidence and negatively correlated with cancer survival rates, consistent with the hypotheses proposed in the introduction. Moreover, the results further validate the importance of race in cancer risk. Our findings align with existing literature in several respects, reinforcing the significant role of SDOH in cancer risk and survival prognosis. The literature consistently indicates that older individuals face a significantly increased risk of developing cancer and shorter survival times following diagnosis.^[22,23]^ A study quantifying risk factors across 21 types of cancer found that men are at a greater risk of developing cancer in most cases.^[24]^ Furthermore, cancer mortality rates are generally higher among male patients compared with females.^[25]^ Several broad racial cancer risk studies have also found that individuals from other racial backgrounds typically have a lower cancer risk than White individuals.^[26,27]^ In addition, individuals with unstable housing are regarded as a high-risk group for cancer.^[28]^ Regarding prognosis, multiple studies have emphasized the necessity of employment support for the survival of cancer survivors, with unemployed cancer survivors generally experiencing shorter survival times.^[29–31]^ A cross-sectional study on colorectal cancer revealed that a better economic status is consistently associated with improved survival outcomes, regardless of tumor location, stage, patient gender, or treatment method.^[32]^ Despite the consistency of our findings with existing literature, there are noteworthy differences in the current research. First, some epidemiological surveys have reported higher cancer incidence rates among females compared with males.^[33]^ Researchers have indicated that in the case of thyroid, gallbladder, and anal cancers, male incidence rates are lower than those of females.^[34]^ Regarding the relationship between race and cancer risk, some studies report that African Americans have a higher incidence rate for all combined malignancies. The reasons for these discrepancies may include: on one hand, the majority of current studies include samples that only cover specific regions and populations, failing to conduct a comprehensive global analysis; on the other hand, while pan-cancer analyses can provide broad conclusions, it remains necessary to conduct stratified discussions of incidence rates across different cancer types. Such stratified analyses can enhance our understanding of how specific population characteristics affect the incidence of particular cancers.

Drawing from relevant biological and sociological theories, it is crucial to investigate the potential mechanisms through which SDOH influence cancer risk and prognosis. Current research focuses on 3 main aspects: environmental impact: researchers have found that individuals experiencing unstable housing, job instability, and low income are more likely to be exposed to harmful factors such as solar radiation, air pollution, and heavy metal exposure, which are typically associated with increased cancer risk.^[35–39]^ Nutrition and lifestyle: among those with unstable housing, ≈70% engage in alcohol abuse, and the prevalence of smoking ranges from 3% to 50%.^[28,40]^ Furthermore, unstable housing interacts with food insecurity.^[41]^ On the other hand, cancer survivors often find themselves in economically toxic conditions such as unemployment and financial instability, which can compel them to adopt unhealthy lifestyles.^[42]^ These poor lifestyle choices are generally considered high-risk factors for cancer.^[43]^ Chronic stress state: surveys indicate that about 50% of individuals with unstable housing suffer from depression and/or anxiety.^[44]^ There is clear evidence that job instability and lower income have direct causal relationships with a range of psychological issues.^[45–47]^ Under such psychological stress, individuals are placed in a state of chronic stress, leading to the activation of the hypothalamic–pituitary–adrenal axis and sympathetic nervous system, which can trigger the onset of cancer.^[48]^

Based on the aforementioned research findings, interventions addressing SDOH hold significant implications for clinical practice and public health management. First, from a clinical perspective, the identification of factors such as older age, male gender, unstable employment, and lower income-to-poverty ratios can assist healthcare providers in better assessing the survival prognosis of cancer survivors. This identification can support the formulation of targeted treatment plans and enhance care services for patients. For example, medical teams can provide mental health assessments and interventions for high-risk patients to alleviate symptoms of depression and anxiety, thereby improving their quality of life and treatment adherence. Second, at the public health level, these findings can bolster the development and implementation of public policies aimed at reducing cancer incidence and improving survival outcomes. For instance, community-level initiatives promoting early screening programs for high-risk populations can enhance primary cancer prevention efforts. In addition, increasing employment and economic support for cancer survivors can significantly improve their quality of life and extend survival prognosis. The following specific examples illustrate how predictive models can be utilized in practice to strengthen cancer risk and prognosis management: Community level: by utilizing demographic data from government public health department databases, alongside data on SDOH collected at the community level, populations can be stratified to identify high-risk cancer groups. Regular cancer screenings can be conducted for these high-risk individuals, and interventions targeting their adverse health social determinants, such as employment guidance, can be implemented. Hospital level: through the collection of data from inpatient medical records or retrospective analyses of electronic medical records for patients with cancer, appropriate follow-up plans can be developed for patients with high-risk factors. Furthermore, lifestyle and nutritional guidance can be improved, and collaborations with government agencies can facilitate the implementation of community intervention measures targeting the adverse health social determinants identified in these patients.

This study has several limitations. First, as a survey based on NHANES, most of the study characteristics are derived from self-reported data from participants, which introduces uncertainty and bias into the results. To enhance the model, it would be beneficial to set clearer questions in the questionnaires, conduct multiple assessments on the same individuals, and incorporate objective hematological and imaging data. Second, the inclusion of samples from a single database limits the external validation and testing of the developed model. Future large-scale prospective studies should be conducted in collaboration with multiple centers, incorporating different characteristics for sensitivity analysis or setting various subgroups, such as different cancer populations, for subgroup analysis to further validate the model. Third, cancer development is a dynamic process; treatment modalities, duration of treatment, and SDOH are often multifaceted and complex throughout this journey. The SHAP value plots from the cancer survival prediction model indicate that the influence of SDOH is significantly lower than that of age. Therefore, it is essential to include additional social determinants, such as social network support or comprehensive clinical data, in future research. Regular follow-up of patients to observe changes in their SDOH and updating the model accordingly is also crucial. Moreover, integrating related disciplines such as biology, sociology, and psychology can help identify the interactions between these factors and SDOH, providing a more comprehensive perspective for model improvement. In summary, to address the limitations and implement improvement measures, future research on SDOH concerning cancer risk and survival prediction models should involve multicenter, large-scale prospective studies. Data collection should include a broader range of clinical information, while data analysis should employ subgroup and sensitivity analyses. Regular updates to the model should be based on the collected data. In terms of intervention measures, it is vital for governments, hospitals, and communities to identify high-risk populations and formulate supportive policies, provide follow-up support, and promote multidisciplinary collaboration. In addition, raising public awareness about SDOH and encouraging regular screening for high-risk populations is essential. Implementing these measures will enhance cancer prevention and prognostic management.

## 5. Conclusion

This study developed machine learning models incorporating SDOH using RF and GBM_Surv to predict cancer risk and survival outcomes effectively. The results of this study highlight the significant connection between SDOH and the occurrence and progression of cancer, introducing a new observational perspective to cancer epidemiology by revealing the different impacts of various SDOH on cancer. In addition, this research emphasizes the existence and effects of inequalities in SDOH. This not only establishes a new guiding direction for the formulation of corresponding public health policies and interventions but also suggests that targeting high-risk populations for intervention can effectively reduce health disparities and promote social equity.

## Acknowledgments

The authors thank the participants and staff of the National Health and Nutrition Examination Survey from 2011 to 2018 for their valuable contributions.

## Author contributions

**Conceptualization:** Jianan Jin.

**Formal analysis:** Jianan Jin, Qi Zheng.

**Software:** Jianan Jin, Shiqi Zhang.

**Supervision:** Jianan Jin.

**Validation:** Jianan Jin.

**Visualization:** Jianan Jin.

**Writing – original draft:** Jianan Jin.

**Writing – review & editing:** Jianan Jin, Shiqi Zhang.

**Data curation:** Qi Zheng, Zhenyu Wang.

**Methodology:** Zhenyu Wang.

**Project administration:** Zhenyu Wang.
